# Biologic Therapy in Head and Neck Cancer: A Road with Hurdles

**DOI:** 10.5402/2012/163752

**Published:** 2012-06-13

**Authors:** Pol Specenier, Jan B. Vermorken

**Affiliations:** Department of Medical Oncology, Antwerp University Hospital, Wilrijkstraat 10, 2650 Edegem, Belgium

## Abstract

The epidermal growth factor receptor (EGFR) is overexpressed in the vast majority of cases of squamous cell carcinoma of the head and neck (SCCHN). A high EGFR expression is associated with an unfavorable prognosis. Cetuximab is a chimeric human/murine IgG1 antibody which binds with high affinity to the EGFR. It is the only targeted agent which got approval for the treatment of SCCHN from the regulatory agencies of Europe and the United States, both in locoregionally advanced disease, in association with radiation, and in recurrent/metastatic disease. The outcome of trials involving other EGFR-directed monoclonal antibodies, that is, zalutumumab and panitumumab, was consistent with the results with cetuximab. However these trials failed to meet their primary endpoint. The results with EGFR-directed tyrosine kinase inhibitors have been disappointing. Other potential targets for treatment in SCCHN include the entire ErbB family, the vascular endothelial growth factor (VEGF) and its receptor (VEGFR), the insulin-like growth factor 1 receptor (IGF-1R), the insulin receptor (IR), histone deacetylases (HDAC), the mammalian target of rapamycin (mTOR), the platelet-derived growth factor receptor (PDGFR), heat-shock protein 90 (HSP90), nuclear factor-kappa B (NF-*κ*B), aurora A or B, and phosphatidylinositol 3-kinase (PIK3CA).

## 1. Introduction

Worldwide, squamous cell carcinoma of the head and neck (SCCHN) is the sixth most common cancer and is diagnosed in more than 600,000 patients each year [1]. A better understanding of its biology has been accompanied by the introduction of a large and rapidly expanding number of targeted agents into its management strategies [2]. Planned and ongoing trials in SCCHN involving targeted agents are summarized in Tables 1, 2, 3, and 4 [3]. Potential targets include the epidermal growth factor receptor (EGFR) and the ErbB family, the vascular endothelial growth factor (VEGF) and its receptor (VEGFR), Insulin-like Growth Factor 1 Receptor (IGF-1R), insulin receptor (IR), histone deacetylase (HDAC), mammalian target of rapamycin (mTOR), platelet-derived growth factor (PDGFR), Heat-shock protein 90 (HSP90), Nuclear factor-kappa B (NF-*κ*B), aurora A or B, phosphatidylinositol 3-kinase (PIK3CA).

## 2. EGFR-Directed Therapies

The Epidermal Growth Factor Receptor (EGFR) belongs to the ErbB family of receptors which includes four members: EGFR/ErbB1, ErbB2/Her2/neu, ErbB3, and ErbB4. EGFR consists of an extracellular N-terminal ligand-binding domain, a hydrophobic transmembrane domain, and a C-terminal intracellular domain, which includes the tyrosine kinase domain and the autophosphorylation sites. The Epidermal Growth Factor Receptor (EGFR) is overexpressed in the vast majority of cases of Squamous Cell Carcinoma of the Head and Neck (SCCHN) [4]. A high EGFR expression and an increased EGFR gene copy number are associated with an unfavorable prognosis [5–7]. Two of the potential EGFR targeting strategies that are currently in use in the treatment of SCCHN are the monoclonal antibodies directed at the extracellular domain of the receptor and the small molecule and ATP-competitive tyrosine kinase inhibitors (TKIs).

### 2.1. EGFR-Directed Monoclonal Antibodies

#### 2.1.1. Cetuximab

Cetuximab is the only targeted agent that got approval by the Food and Drug Administration and the European Medicines Agency for the treatment of SCCHN [8, 9] and is still under active investigation in this disease [3] (Tables 1 and 2). Cetuximab is a chimeric human/murine IgG1 antibody which binds with higher affinity to the EGFR than the natural ligands EGF and TNF-*α*, thereby disrupting EGFR signaling pathways. Another mechanism that contributes to the antitumor activity of cetuximab is depletion of the targeted receptors from the cell surface. The availability of EGFR on the cell surface is reduced, and the receptor is downregulated [10, 11]. Finally, cetuximab's construction on an IgG1 framework potentially allows the drug to mediate antibody-dependent cell-mediated cytotoxicity (ADCC) via natural killer (NK) cells and macrophages [12]. Cetuximab is administered once a week at an initial loading of 400 mg/m² followed by a weekly dose of 250 mg/m² [8]. The recommended dose was used in the cetuximab studies in SCCHN studies mentioned below, unless stated otherwise.

### 2.2. Cetuximab in Locoregionally Advanced SCCHN

Bonner et al. [13, 14] conducted a multinational, randomized study comparing radiotherapy alone with radiotherapy plus cetuximab in 424 patients with stages III or IV, nonmetastatic, measurable squamous cell carcinoma (SCC) of the oropharynx, hypopharynx, or larynx. Investigators were required to select one of three radiotherapy-fractionation regimens before patient registration: 70.0 Gy in 35 daily fractions of 2.0 Gy/fraction, 5 fractions/week for 7 weeks, or two daily fractions of 1.2 Gy/fraction up to 72.0–76.8 Gy in 60–64 fractions, 10 fractions/week for 6–6.5 weeks, or a concomitant boost regimen (72.0 Gy in 42 fractions: 32.4 Gy; 1.8 Gy/fraction, 5 fractions/week for 3.6 weeks followed by a morning dose of 21.6 Gy in fractions of 1.8 Gy, 5 fractions/week for 2.4 weeks and an afternoon dose of 18.0 Gy in fractions of 1.5 Gy, 5 fractions/week for 2.4 weeks). Uninvolved nodal areas of the neck were treated with 50 to 54 Gy, depending on the fractionation regimen used. Gross nodal disease received the same dose as the primary tumor. In the group assigned to receive radiotherapy plus cetuximab, cetuximab was initiated one week before radiotherapy at a loading dose of 400 mg/m², followed by a weekly dose of 250 mg/m² for the duration of radiotherapy. The median duration of locoregional control (primary endpoint) was 24.4 months in patients treated with cetuximab plus radiotherapy and 14.9 months in patients treated with radiotherapy alone (hazard ratio (HR) for locoregional progression or death, 0.68; *P* = 0.005). The one-, two-, and three-year rates of locoregional control achieved with radiotherapy plus cetuximab (63, 50, and 47%), were significantly higher than those achieved with radiotherapy alone (55, 41, and 34%, resp.). Median overall survival (OS) for patients treated with cetuximab and radiotherapy was 49.0 months versus 29.3 months in the radiotherapy-alone group (HR for death: 0.73; *P* = 0.018). Grade ≥2 rash was associated with an improved survival [13]. In this pivotal trial, the addition of cetuximab did not lead to an increased incidence of radiation dermatitis. However, as there is only one randomized phase III trial with cetuximab-based bioradiation as opposed to the abundance of data supporting cisplatin-based concurrent chemoradiation (CRT) [15, 16], the latter continues to represent the standard of care for medically fit patients with locoregionally (LA) SCCHN, who can tolerate platinum-based therapy. The addition of cetuximab to cisplatin-based CRT does not further improve the outcome. In Radiation Therapy Oncology Group (RTOG) 0522 [17], 895 evaluable patients with stage III/IV nonmetastatic SCCHN were randomized between chemoradiation (72 Gy in 42 fractions over 6 weeks plus cisplatin 100 mg/m² on days 1 and 22) or the same regimen plus weekly cetuximab. At the time of the third interim analysis after 337 events and after a median followup of 2.4 years for the surviving patients, the conditional power of the trial becoming positive was below 10%, triggering early reporting. Over 90% of the patients received the planned two doses of cisplatin in both arms. The 2-year progression-free survival (PFS), (primary endpoint) was 64.3% with chemoradiation and 63.4% with chemoradiation plus cetuximab (HR: 1.05; 95% confidence interval (CI): 0.84–1.29; *P* = 0.67). The 2-year OS was 79.7 and 82.6%, respectively (HR: 0.87; 95% CI: 0.66–1.15; *P* = 0.17). The estimated 2-year locoregional relapse rate was 19.8 and 24.5%, respectively (*P* = 0.92). The 2-year distant metastasis rate was 12 and 7.6%, respectively (*P* = 0.07). Overall, there was no difference regarding acute grade 3/4 acute toxicities between both arms. However, grade 3/4 mucositis (43 versus 33%) and in-field dermatitis (25 versus 15%) was more common with the addition of cetuximab. Grade 3/4 dermatitis outside the radiation field occurred in 19% of the patients treated with cetuximab.

The TREMPLIN trial [18] is a randomized phase II study in patients with SCC of the larynx or hypopharynx suitable for total laryngectomy. After three 3 weekly cycles of TPF (docetaxel 75 mg/m² and cisplatin 75 mg/m² on day 1 followed by 5-FU 750 mg/m²/day, days 1–5), patients who obtained at least a partial response (82% of the patients) were randomized to receive radiotherapy (70 Gy in 35 fractions over 7 weeks) either with cisplatin 100 mg/m² on days 1, 22, and 43 or with weekly cetuximab. The treatment compliance was better in the cetuximab arm with 71% of the patients receiving all planned cetuximab administrations. Forty-three percent of the patients received three cycles of cisplatin, and 83% received 2 cycles. There was no difference in grade 3/4 mucosal toxicity, but grade 3/4 in-field dermatitis was more frequently observed with cetuximab (57 versus 26%; *P* < 0.001). Grade 1 renal dysfunction at last evaluation was observed in 22.4% of the patients treated with cisplatin. The larynx preservation rate 3 months after treatment (primary endpoint) was 95% with cisplatin versus 93% with cetuximab. The locoregional failure rate after a median followup of 36 months was 11.7% with cisplatin and 21.4% with cetuximab. However, more salvage laryngectomies were performed in the cetuximab arm, resulting in a similar ultimate locoregional failure rate in the two arms (10% versus 8.9%). The 2-year laryngoesophageal dysfunction-free survival was 79% with cisplatin versus 71% with cetuximab (*P* = 0.3).

Seiwert et al. [19] randomized 110 patients with LA SCCHN, who had received 2 cycles of carboplatin, paclitaxel, and cetuximab as induction chemotherapy, between weekly cetuximab in combination with either 5-FU, hydroxyurea, and hyperfractionated week-on week-off radiotherapy (72–74 Gy) (CetuxFHX), or cisplatin, accelerated radiation with concomitant boost (CetuxPX) (72 Gy). After a median followup of 21 months, 2-year OS rates were 89.5% with CetuxFHX and 91.4% with CetuxPX arm. Two-year PFS rates were 82.3% and 89.7%, respectively (*P* = 0.18). Grade ≥ 3 mucositis was present in 91.1% (CetuxFHX) and 94.3% (CetuxPX) of patients; grade ≥ 3 dermatitis in 82.1% and 50.9%, respectively. Ninety-five percent of patients completed therapy, demonstrating that cetuximab can be incorporated safely in both CRT platforms.

Argiris et al. [20] enrolled 32 patients in a phase I study combining pemetrexed, cetuximab and radiotherapy in poor prognosis head and neck cancer. Cohort A included patients who had not been previously irradiated, while cohort B included patients who had received prior irradiation. Pemetrexed was administered on days 1, 22, and 43. The maximum tolerated dose (MTD) of pemetrexed was 500 mg in cohort A and 350 mg/m² in cohort B. Grade 3/4 neutropenia was common (50% in cohort A and 33% in cohort B) and febrile neutropenia was the most frequent doselimiting toxicity. Prophylactic antibiotics are recommended. Grade 3/4 mucositis was observed in 8 of the 9 patients treated at the MTD in cohort A [20].

In Eastern Cooperative Oncology Group (ECOG) E2303 [21], 63 patients with resectable stage III/IV SCCHN, were treated with 6-week cycles of paclitaxel, carboplatin at an AUC of 2 and cetuximab, followed by CRT (weekly paclitaxel, 30 mg/m², carboplatin AUC 1, and cetuximab). If at week 14, after a radiation dose of 50 Gy, tumor was still present on biopsy, salvage surgery was performed. In case of a negative biopsy (91% of the patients), CRT was continued to a total dose of 68–72 Gy. Two-year PFS and OS rate were 82 and 66%, respectively. A local recurrence occurred in 17.5% of the patients. Jordan et al. [22] treated 152 T3-T4 SCCHN patients with three 3-week cycles of TPF (docetaxel 75 mg/m² on day 1, cisplatin 35 mg/m² on days 1 and 2, and 5-FU 750 mg/m²/day, as a continuous infusion, days 1–5, with pegfilgrastim support) followed by chemoradiation (63 Gy in 35 fractions of 1.8 Gy over 7 weeks and weekly cisplatin, 40 mg/m²) plus weekly cetuximab. The complete response rate in the 142 patients, who were evaluated after the completion of therapy, was 57%. Grade 3/4 toxicities occurred in 34/142 patients (24%). In 15 patients, CRT had to be interrupted due to dermatitis.

#### 2.2.1. Maintenance Treatment

Ferris et al. [23] treated 34 LA SCCHN patients with three 3-weekly cycles of cisplatin 75 mg/m² and docetaxel 75 mg/m² plus weekly cetuximab followed by CRT (70 Gy in 2 Gy fractions over 7 weeks, weekly cisplatin 30 mg/m²) plus weekly cetuximab, followed by weekly cetuximab maintenance for 6 months. The 3-year PFS rate was 70%.

Mesia et al. [24] studied the role of cetuximab maintenance therapy in patients with LA SCC of the oropharynx. Ninety-one patients were randomized. Patients in group A were treated concomitant radiotherapy, 69.9 Gy in 28 days, plus weekly cetuximab. Patients in group B received an additional 12-week administrations of cetuximab. The locoregional control rate at 1 year (primary endpoint) was 56.8% with bioradiation and 60.5% with bioradiation followed by cetuximab maintenance. At 1 year, event-free survival rates were 55.6 and 60.9%, respectively, and OS rates were 75.6 and 87%, respectively.

### 2.3. Cetuximab in Recurrent/Metastatic SCCHN

#### 2.3.1. First-Line Treatment

In the EXTREME trial (Erbitux in First-Line Treatment of Recurrent or Metastatic Head and Neck Cancer) [25], 442 patients with previously untreated R/M SCCHN were randomized to receive cisplatin 100 mg/m² or carboplatin at an area under the curve (AUC) of 5 mg/mg/min as an 1-hour infusion, followed by 5-FU 1000 mg/m² day for 4 days as a continuous infusion every 3 weeks for a maximum of 6 cycles, either alone or in combination with cetuximab. The use of cisplatin or carboplatin was at the discretion of the investigator. After a maximum of six cycles of chemotherapy, patients in the cetuximab group who had at least stable disease received cetuximab monotherapy until disease progression or unacceptable toxic effects, whichever occurred first. Cross-over was not allowed. The median overall survival (primary endpoint) was 10.1 months (95% CI: 8.6–11.2) in the cetuximab group and 7.4 months (95% CI: 6.4–8.3) in the chemotherapy-alone group (HR for death: 0.80; 95% CI: 0.64–0.99; *P* = 0.04). The median followup was 19.1 months in the cetuximab group and 18.2 months in the chemotherapy-alone group. Median PFS was 5.6 months and 3.3 months for the combined group and the chemotherapy alone group, respectively (HR for progression: 0.54; 95% CI: 0.43–0.67; *P* < 0.001). The overall response rate (ORR) was 36 and 20%, respectively (odds ratio (OR): 2.33; *P* < 0.001), and the disease control rate (DCR) was 80 and 60%, respectively (OR: 2.88; *P* < 0.001). Among the 100 patients who received cetuximab as maintenance treatment, the median PFS was 12 weeks from the start of maintenance treatment. The safety profile of the study treatment was consistent with that expected for the agents used, with no significant difference in the incidence of grades 3 or 4 adverse events between treatment arms. However, there were 9 cases of sepsis in the cetuximab group, as compared with 1 case in the chemotherapy-alone group (*P* = 0.02), and there were 11 cases of hypomagnesemia in the cetuximab group, as compared with 3 cases in the chemotherapy-alone group (*P* = 0.05). Grade 3 skin reactions were seen in 9% of the patients who received cetuximab. There were four grade 3 and two grade 4 infusion-related reactions after cetuximab [25]. ECOG [26] randomized 117 R/M SCCHN patients to receive cisplatin 100 m/m² every 4 weeks plus either weekly cetuximab (group A) or placebo (group B). Primary endpoint was PFS. Median PFS was 4.2 months in arm A and 2.7 in arm B (HR: 0.78; 95% CI: 0.54–1.12; *P* = 0.09). Median OS was 9.2 months in arm A and 8.0 months in arm B (*P* = 0.21). The ORR was 26% and 10%, respectively (*P* = 0.03) [26].

Hitt et al. [27] treated 46 patients with weekly cetuximab and paclitaxel 80 mg/m² as first-line regimen for recurrence of metastatic SCCHN. The ORR and DCR were 54 and 80%, respectively. Median PFS and OS were 4.2 and 8.1 months, respectively. Common grade 3/4 adverse events were acne-like rash (24%), asthenia (17%), and neutropenia (13%) [27]. In Groupe d'Oncologie Radiothérapie Tête Et Cou (GORTEC) 2008–03 [28], 54 patients who had not received prior chemotherapy for R/M SCCHN were treated with docetaxel 75 mg/m² and cisplatin 75 mg/m² every 3 weeks and weekly cetuximab, up to 4 cycles, followed by cetuximab maintenance in the absence of disease progression. Patients received prophylactic lenograstim. The ORR and DCR were 51.9 and 96.1%, respectively. At time of reporting, 65% of the patients were still alive, and median OS exceeded 11 months. High EGFRvIII and amphiregulin expression levels identify SCCHN patients who are less likely to benefit from combination treatment with cetuximab and docetaxel [29].

#### 2.3.2. Second-Line Treatment

Three phase II trials examined the role of cetuximab in platinum-refractory or platinum-resistant disease [30–33]. Responses (10–13%) were observed irrespective of reintroducing the originally used platinum compound to which they had become refractory or resistant. The survival of around 6 months achieved with cetuximab in platinum-refractory disease was found similar to that seen in first-line therapy in R/M-SCCHN and represented an increase in survival of 2.5 months compared with platinum-refractory historical controls [34]. Based on these results and particularly considering the fact that about 50% of the patients showed disease control, cetuximab monotherapy seems to be an option for patients with R/M SCCHN who have progressed on platinum-based chemotherapy.

Fury et al. [35] randomized 61 patients, who had received ≤2 prior cytotoxic chemotherapy regimens for R/M SCCHN to receive cetuximab every 2 weeks at either 500 (A) or 750 mg/m² (B). The ORR was 11% in both arms. PFS was also similar (65 days versus 57 days). Median OS was 8.1 months. Cetuximab 500 mg/m^2^ every 2 weeks seemed to yield similar efficacy and tolerability as conventional weekly dosing for patients with R/M SCCHN. However, it is unclear how many of the patients in this study had platinum-refractory disease. There is no apparent efficacy advantage associated with dose escalation to 750 mg/m^2^ Q2W.

#### 2.3.3. Zalutumumab

Zalutumumab is a human IgG1 monoclonal antibody targeting EGFR. Machiels et al. [36] randomly allocated 286 eligible incurable SCCHN in a 2 : 1 ratio to receive either zalutumumab plus best supportive care (zalutumumab group) or best supportive care with optional methotrexate (control group). Eligible were patients with progressive disease according to RECIST confirmed by an independent review committee during or within 6 months after the failure of platinum-based chemotherapy (at least two cycles of cisplatin [≥60 mg/m² per cycle] or carboplatin [≥250 mg/m² per cycle] with an interval between the cycles of <4 weeks). Also eligible were patients with platinum intolerance which was defined as discontinuation or dose reduction of platinum-based chemotherapy due to adverse or toxic effects, irrespective of response. The dose of zalutumumab was titrated according to rash. Patients were given an initial loading dose of 8 mg/kg followed up by two week doses of 4 mg/kg by intravenous infusion in 1 h. After the first three administrations, in patients with no rash or grade 1 rash, the dose was increased by 4 mg/kg every 2 weeks up to a maximum dose of 16 mg/kg. Patients with grade 2 rash remained at the same dose. Treatment was withheld from patients with grade 3 rash until rash resolved to grade 1. Seventy-two percent of the control patients received methotrexate from the initiation of the trial, and a further 6% started methotrexate during the trial. Median OS (primary endpoint) was 6.7 months (95% CI: 5.8–7.0) in the zalutumumab group and 5.2 months (95% CI: 4.1–6.4) in the control group (hazard ratio (HR) for death, stratified by WHO performance status: 0.77; 97.06% CI: 0.57–1.05; unadjusted *P* = 0.0648). Progression-free survival was longer in the zalutumumab group than in the control group (HR for progression or death, stratified by WHO performance status: 0.63; 95% CI: 0.47–0.84; *P* = 0.0012). The most common grade 3/4 adverse events were rash (21% of patients in the zalutumumab group versus none in the control group), anemia (6% and 5%, resp.), and pneumonia (5% and 2%, resp.). Grade 3/4 infections occurred in 15% of the patients in the zalutumumab group and 9% in the control group [36].

#### 2.3.4. Panitumumab

Panitumumab is a fully human IgG2 EGFR-directed antibody. Its pharmacokinetic profile allows a convenient three-week administration. In the SPECTRUM trial (Study of Panitumumab Efficacy in Patients With Recurrent and/or Metastatic Head and Neck Cancer), [37] 657 patients were randomized between cisplatin 100 mg/m² on day 1, followed by 5-fluorourcacil 1000 mg/m²/day for 4 days or the same chemotherapy plus panitumumab 9 mg/kg administered on day 1. Cycles were repeated every 3 weeks up to a maximum of 6 cycles. Patients receiving panitumumab without progression after 6 cycles could continue panitumumab monotherapy until progression. Patients were allowed to switch from cisplatin to carboplatin (AUC 5) during treatment for specific cisplatin-related toxicities, such as grade ≥2 neurotoxicity or a drop in creatinine clearance to <50 mL/min. Overall survival was the primary endpoint. The median OS in the combined arm was 11.1 months compared to 9.0 months in the chemotherapy alone arm (HR = 0.87; 95% CI: 0.73–1.05; *P* = 0.14). However, there was a statistically significant difference in response rate (36% versus 25%; *P* = 0.007) and PFS (median 5.8 months versus 4.6 months; HR = 0.78; 95% CI: 0.66–0.92; *P* = 0.004) in favour of the panitumumab-containing arm. Although the SPECTRUM trial failed to meet its primary endpoint, the results are nevertheless consistent with the outcome of the EXTREME trial.

#### 2.3.5. Nimotuzumab

Nimotuzumab is a humanized EGFR targeting monoclonal antibody which was studied in multiple phase II trials. A weekly fixed dose of 200 mg was established as recommended dose for use in combination with radiotherapy in LA SCCHN. No grade 3/4 adverse events were reported in a pilot study of weekly nimotuzumab (200 mg), radiotherapy (66 Gy in 33 fractions/2 Gy per fraction over 6.5 weeks) and weekly cisplatin (40 mg/m²) in 17 patients with LA SCCHN [38]. Rodríguez et al. [39] enrolled 106 patients with inoperable LA SCCHN in a double blind, randomized phase II trial. The primary endpoint of the trial was the complete response rate, which was 59.5% with nimotuzumab versus 34.2% with placebo (*P* = 0.028). Babu et al. [40] also conducted a randomized phase II trial in patients with LA SCCHN. Ninety-two patients were enrolled, and 76 were evaluable. Patients included in group A were treated with radiation (60–66 Gy) with or without nimotuzumab. At 48 months of followup, OS was 34% with RT plus nimotuzumab versus 13% with radiotherapy alone.

Patients included in group B were treated with CRT (60–66 Gy plus weekly cisplatin at a dose of 50 mg) with or without weekly nimotuzumab. At 48 months of followup, OS rate was 47% with CRT plus nimotuzumab versus 21% with CRT (*P* = 0.01).

### 2.4. Tyrosine Kinase Inhibitors

Tyrosine kinase inhibitors which have been tested in SCCHN include gefitinib and erlotinib, which are reversible specific EGFR TKIs, lapatinib, a reversible dual EGFR/Her2 TKI, afatinib, an irreversible dual EGFR/Her2 TKI, and PF-00299804, a potent irreversible pan-HER TKI.

#### 2.4.1. Gefitinib and Erlotinib

**Recurrent/Metastatic SCCHN**

Argiris et al. [41] planned to randomize 330 patients to receive docetaxel 35 mg/m² on days 1, 8, and 15 every 28 days either plus placebo or in combination with gefitinib 250 mg/day. The data monitoring committee recommended early stopping of enrollment after inclusion of 270 patients because there was <5% chance to meet the primary endpoint (overall survival). Eligible were patients who were previously treated with chemotherapy for R/M SCCHN (73% of the patients) and patients previously untreated for R/M SCCHN either with a poor performance status (ECOG 2) or in case of relapse within 6 months after chemotherapy given as part the primary treatment with curative intent. Median OS was 6.8 months with docetaxel plus placebo versus 6.2 months with docetaxel plus gefitinib (HR 0.99; 95% CI: 0.75–1.31; *P* = 0.97). The time to progression was significantly longer with the addition of gefitinib (median 3.5 months versus 2.1 months; HR 0.69; 95% CI: 0.49–0.99; *P* = 0.047). In the IMEX trial [42], 486 R/M SCCHN patients were randomly assigned to oral gefitinib 250 mg/day, gefitinib 500 mg/day, or methotrexate 40 mg/m² intravenously weekly. Physicians and patients were blinded to the gefitinib dose. Two coprimary analyses compared OS between each gefitinib dose and methotrexate. Patients were stratified into two groups: group A (*n* = 256) consisted of patients who had stable or progressive disease after at least two cycles of platinum-based chemotherapy for recurrent disease; group B (*n* = 230) consisted of patients who were considered unsuitable for platinum-containing chemotherapy. Neither gefitinib 250 mg/day nor gefitinib 500 mg/day improved OS compared with methotrexate (HR: 1.22; 95% CI: 0.95–1.57; *P* = 0.12; HR: 1.12; 95% CI: 0.87–1.43; *P* = 0.39, resp.). Median OS was 5.6, 6.0, and 6.7 months in the gefitinib 250 mg/day, gefitinib 500 mg/day, and methotrexate groups, respectively. In group A, OS was significantly longer with methotrexate (HR for death: gefitinib 250 mg versus methotrexate: 1.62; *P* = 0.01; gefitinib 500 mg versus methotrexate: 1.5; *P* = 0.02). Tumor hemorrhage-type events were more common with gefitinib (250 and 500 mg) than with methotrexate (8.9%, 11.4%, and 1.9%, resp.). The most common adverse events were rash, diarrhea, cancer pain, nausea, and vomiting with gefitinib, and stomatitis, nausea, and constipation with methotrexate.

The OS with gefitinib in the IMEX trial was similar to what was observed in earlier uncontrolled phase II studies with gefitinib or erlotinib [43–46]. Dose escalation of gefitinib adaptive to skin toxicity grade did not improve response rate in a phase II trial conducted by Perez et al. [47].

**Locoregionally Advanced SCCHN**

William Jr. et al. [48] randomized 34 patients with resectable SCCHN to receive erlotinib daily for 2 to 8 weeks prior to surgery at standard (150 mg) or high doses (200 mg in never/former smokers, and 300 mg in current smokers). There were no grade 4 toxicities or erlotinib-related surgical complications. Response rates were documented in 18% and 29% of the patients in the low-and high-dose arms, respectively, suggesting that a subgroup of previously untreated SCCHN is highly sensitive to EGFR tyrosine kinase inhibition.

Hayes et al. [49] randomly assigned 128 patients with LA SCCHN to receive either cisplatin 100 mg/m² on days 1, 22, and 43 combined with 70 Gy of radiotherapy (arm A) or the same treatment plus 150 mg of erlotinib starting one week before CRT and continued until the completion of radiotherapy (arm B). Serious adverse events were observed in 33% and 32% of the patients in arm A and B, respectively. Most common serious adverse events were nausea, vomiting, and dehydration and accounted for 30% of all SAEs reported, with no difference between arms. Efficacy data are pending. Gregoire et al. [50] enrolled 226 patients in a randomized phase II trial testing the value of gefitinib during and/or after CRT in LA SCCHN. Patients received either placebo during CRT followed by adjuvant placebo, or gefitinib 250 mg or 500 mg/day during CRT followed by placebo, gefitinib 250 mg or 500 mg/day during and after CRT, or placebo during CRT followed by adjuvant gefitinib at a dose of 250 mg or 500 mg/day. Adjuvant therapy was administered for a maximum of 2 years. There was no difference in 2-year local disease control rate (primary endpoint) between the 7 treatment arms. Soulieres et al. [51] evaluated the toxicity and recommended dose for adjuvant erlotinib after definitive CRT for LA SCCHN. No dose limiting toxicities were observed at a daily dose of 100 or 150 mg for 6 months. At the 150 mg dose, 46% of the patients received ≥90% dose intensity. The approach can be considered for a phase III trial.

#### 2.4.2. Dual EGFR/Her2- and Pan HER-Inhibitors

Encouraging preliminary results in R/M SCCHN after failure of a platinum-containing therapy were reported with afatinib, a dual EGFR/Her2 irreversible tyrosine kinase inhibitor, which was compared to single-agent cetuximab in a randomized phase II study. The overall response rate with afatinib compared favorably to the ORR with cetuximab [52]. Harrington et al. [53] enrolled 67 patients into a randomized, placebo-controlled, phase II trial, exploring the potential benefit of adding lapatinib to CRT in patients with LA SCCHN. Lapatinib (1500 mg/day) or placebo were started 1 week before CRT (70 Gy in 35 fractions over 7 weeks plus cisplatin 100 mg/m² on days 1, 22, and 43) and continued during and after CRT until disease progression. The addition of lapatinib did not impair the timely administration of CRT and did not lead to an increase in mucositis and radiation dermatitis. The complete response rate at 6 months after treatment (primary endpoint) was 36% with placebo and 53% with lapatinib. The PFS rates at 12 months were 45 and 59%, respectively. Del Campo et al. [54] treated 107 patients with LA SCCHN with either lapatinib or placebo for 2–6 weeks prior to CRT. The overall response rate before CRT in the 40 patients who received >4 weeks of lapatinib was 17%.

Siu et al. [55] enrolled 69 patients in a phase II study with PF-00299804 at a dose of 45 mg QD, in previously untreated R/M SCCHN. Grade 3 adverse events were diarrhea (16%), fatigue (9%), acneiform dermatitis (7%), and hand-foot reaction (4%). The ORR and DCR were 12.7 and 60%, respectively. Median PFS and OS were 2.8 and 8.3 months, respectively.

## 3. VEGFR-Directed Therapies

A meta-analysis conducted by Kyzas et al. [56] demonstrated that VEGF protein overexpression, as detected with immunohistochemistry, is associated with a worse OS in patients with SCCHN.

### 3.1. Bevacizumab

Bevacizumab is a humanized IgG1 monoclonal antibody that binds selectively to all isoforms of human VEGF and neutralizes the biologic activities of VEGF by blocking the binding of VEGF to its receptors on the surface of endothelial cells. Bevacizumab enhances the activity of chemotherapy in SCCHN xenografts [57, 58].

Argiris et al. [59] combined pemetrexed 500 mg/m² and bevacizumab 15 mg/kg given intravenously every 21 days until disease progression in 40 patients presenting with previously untreated R/M SCCHN. The median TTP (primary endpoint) was 5 months, and the median OS was 11.3 months. In 37 evaluable patients, the ORR was 30%, and the DCR was 86%. Grade ≥ 3 bleeding events occurred in 6/40 patients (15%) and was fatal in 2 (5%) [59]. Bevacizumab, 10 mg/kg, administered on day 1 of each 2-week cycle, can be safely combined with FHX CRT regimen, consisting of five 2-week cycles of hydroxyurea 500 mg orally bid, 5-FU 600 mg/m²/day administered as a continuous infusion, and radiotherapy, 1.5 Gy bid for 5 days followed by 9 days without therapy (FHX), in patients with poor prognosis SCCHN [60]. Salama et al. [61] conducted a randomized phase II study evaluating the impact of adding bevacizumab (B) to the FHX regimen. Eligible were patients with T1-3, N0-1 and T4, N0-1 SCCHN. The study was terminated early after enrollment of 26 patients following unexpected locoregional progression. All locoregional progression occurred in T4 tumors randomized to BFHX. Two patients receiving BFHX died during therapy, and one died shortly after therapy. Two-year OS was 89% in patients treated with FHX and 58% in patients treated with BFHX. These unexpected findings are not well understood and could be due to chance, given the small sample size. Harari et al. [62] demonstrated the safety and feasibility of combining CRT (70 Gy in 33 fractions and weekly cisplatin, 30 mg/m²) with bevacizumab weeks −3, 1, 4, and 7 with dose escalation from 5 to 10 to 15 mg/kg in 10 patients with LA SCCHN. Several patients manifested tumor regression following administration of bevacizumab alone. No dose-limiting toxicities (DLTs) were observed.

### 3.2. Tyrosine Kinase Inhibitors

Sorafenib and sunitinib are oral inhibitors of multiple kinases including the receptor tyrosine kinases of vascular endothelial growth factor (VEGF) receptor. Phase II data with both agents in R/M SCCHN are extremely disappointing. Elser et al. [63] treated 27 patients with R/M SCCHN or nasopharyngeal carcinoma (7 patients), who had received ≤1 chemotherapy for recurrent or metastatic disease with sorafenib 400 mg twice daily on a continuous basis. The treatment was well tolerated with few grade 3 or 4 toxicities. However, anticancer activity was modest. One patient achieved a partial response (3.7%). Disease control rate was 40.7%. The median TTP was 1.8 months, and median OS was 4.2 months. The same regimen was evaluated in the Southwest Oncology Group study S0420 [64], which enrolled 41 R/M SCCHN patients who had not received prior chemotherapy for R/M disease. The overall confirmed response rate was a mere 2%. However, the estimated median PFS was 4 months, and the estimated median OS was 9 months. In the GORTEC 2006–01 study [65], 38 patients with R/M SCCHN received sunitinib 37.5 mg/day on a continuous basis. Forty-five percent and 3% of the patients had received one and two prior chemotherapy regimens for R/M disease, respectively. Local complications, including the appearance or worsening of tumor skin ulceration or tumor fistula, were recorded in 39.5% of the patients, and a fatal arterial bleeding in the head and neck region occurred in 10.5% of the patients. The ORR and DCR were 2.6 and 50%, respectively. Median PFS and OS were 2 and 3.4 months, respectively. Fountzilas et al. [66] treated 17 R/M SCCHN patients with sunitinib 50 mg per day administrated in 4-week cycles followed by a rest period of 2 weeks as first-line treatment for R/M disease and observed no objective responses. Disease control rate was 18%, median TTP was 2.3 months, and median OS was 4 months. The same intermittent sunitinib regimen was used by Choong et al. [67] who observed an ORR of 4.5% and a DCR of 27.3%. Sabichi et al. [68] combined paclitaxel, carboplatin AUC 6, administered every 3 weeks, and sorafenib 400 mg bid, days 2–19, in patients with R/M SCCHN. Twenty-eight patients were enrolled and 22 were evaluable for response. The ORR and DCR were 68 and 91%, respectively. Vandetanib is an inhibitor of vascular endothelial growth factor receptor-2 (VEGFR-2), EGFR, and rearranged during transfection (RET) tyrosine kinases. It restores HNSCC cells' sensitivity to cisplatin and radiation in vitro and in vivo [69]. As a single agent, it has antiproliferative effects on SCCHN cells in vitro and inhibits tumor growth in nude mice orthotopically injected with human SCCHN cells [70]. Vandetanib can be safely combined with radiotherapy (2.2 Gy/d, 5 days/week up to a total dose of 66 Gy) or radiotherapy (2 Gy/d, 5 days/week up to a total dose of 70 Gy) plus weekly 30 mg/m² of cisplatin [71].

## 4. Other Targets

### 4.1. Heat-Shock Protein 90

Heat-shock protein 90 (HSP90) is a protein which chaperones multiple client oncoproteins involved in tumor progression. Okui et al. [72] investigated the antitumor effect of the novel HSP90 inhibitor NVP-AUY922 against oral SCC (OSCC). NVP-AUY922 inhibited the proliferation of OSCC cells in vitro and induced a robust antitumor response and suppressed p-Akt and VEGF expression in an HSC-2 xenograft model.

### 4.2. Sirtuin

Several sirtuin family members (SIRT1-7) function either as nicotinamide adenine dinucleotide (NAD)-dependent deacetylases or as ADP-ribosyl transferases and are involved in carcinogenesis [73]. Alhazzazi et al. [74] demonstrated that SIRT3 is overexpressed in OSCC in vitro and in vivo and that SIRT3 downregulation inhibits OSCC cell growth and proliferation and increased OSCC cell sensitivity to radiation and cisplatin treatments in vitro.

### 4.3. Histone Deacetylase

Histone deacetylases are enzymes involved in remodeling of chromatin by deacetylating the lysine residues and play a pivotal role in epigenetic regulation of gene expression. There is extensive preclinical evidence supporting the testing of HDAC inhibitors in SCCHN. HDAC inhibitors have radio-enhancing properties [75, 76], increase the susceptibility of SCC cell lines to cisplatin in vitro [77, 78], and inhibit tumor growth in xenograft models of SCCHN [79]. Bruzzese et al. [80] demonstrated that the histone deacetylase inhibitor vorinostat in combination with the EGFR tyrosine kinase inhibitor gefitinib induced synergistic inhibition of proliferation, migration, and invasion as well as induction of apoptosis in SCCHN cells, including cells resistant to gefitinib.

### 4.4. Aurora A and B

High expression of aurora A or B is associated with a worse outcome in SCCHN [81–86].

Hoellein et al. [87] combined a dual aurora A/aurora B inhibitor with cetuximab in SCCHN cell lines in vitro and observed at least an additive effect. Aurora kinase inhibition was able to overcome resistance to cetuximab [87].

### 4.5. Mammalian Target of Rapamycin

Preclinical data strongly support the testing of mammalian target of rapamycin (mTOR) in SCCHN [88–90]. Activation of mTOR is observed in the majority of SCCHN [91] and is associated with a poor outcome [92]. Patel et al. [93] found that inhibition of mTOR diminished lymphangiogenesis in the primary tumors and prevented the dissemination of SCCHN cancer cells to the cervical lymph nodes in an orthotopic mouse model [93]. Temsirolimus enhances the growth-inhibiting effects of the combination of bevacizumab, cetuximab, and irradiation in head and neck cancer xenografts [94]. Aissat et al. [95] demonstrated that rapamycin displays antiproliferative effects and induces apoptosis in SCCHN cell lines and that combination of rapamycin with paclitaxel or carboplatin displayed synergistic and additive effects [95]. Temsirolimus appeared to be a more potent radiosensitizer than cisplatin in mice bearing squamous cell carcinoma xenografts [96]. In a pharmacodynamic evaluation of temsirolimus in SCCHN patients, Ekshyyan et al. found a significant inhibition of the mTOR pathways in tumor cells and in peripheral blood mononuclear cells [97]. Everolimus 10 mg/day, days 1–21 can be safely combined with cisplatin 20 mg/m², days 1, 8, and 15 of a 28-day cycle in patients with solid tumors [98].

### 4.6. c-Src

c-Src is a nonreceptor cytoplasmic tyrosine kinase that regulates signals from cell surface molecules and that plays a key role in modulating multiple cellular functions by activating the signal transducer and activator of transcription (STAT) family of transcription factors. Although preclinical data provided a rationale for testing c-Scr inhibitors in SCCHN, the outcome with single-agent c-Scr inhibitors in patients with R/M SCCHN was disappointing. Brooks et al. [99] treated 15 R/M SCCHN patients with dasatinib, a potent inhibitor of Src-family kinases EphA2, at a dose 100 mg twice daily. No objective responses were observed and the median PFS was less than 1 month. Saracatinib is also an orally available Src kinase inhibitor which was administered to 9 R/M SCCHN patients at a daily dose of 175 mg [100]. Eight patients had disease progression within the first eight-week cycle, and one patient was removed from the study after 11 days due to rapid clinical decline. The study was closed early due to lack of efficacy according to the early stopping rule [100].

### 4.7. Phosphatidylinositol-3-Kinase

Rampias et al. [101] detected HRAS mutations in 29% of 105 SCCHN specimens. Four percent of the specimens harbored PIK3CA mutations. Cell lines bearing HRAS or PIK3CA mutations are resistant to cetuximab. This resistance can be overcome by addition of a PI3K inhibitor.

### 4.8. Nuclear Factor Kappa B

Nuclear factor kappa B is overexpressed in SCCHN, and NF-*κ*B expression is associated with a poor prognosis. Bortezomib is a small-molecule proteasome inhibitor which affects multiple signaling pathways including NF-*κ*B. Chung et al. treated 25 R/M SCCHN patients with bortezomib 1.6 mg/m² and docetaxel 40 mg/m² on days 1 and 8 of a 21-day cycle and observed a ORR and DCR of 5 and 52%, respectively.

## 5. Combination of Targeted Agents

Cohen et al. [102] combined erlotinib 150 mg/day and bevacizumab IV every 3 weeks. As no dose-limiting toxicities were observed in the phase I portion of the study which included 10 patients up to the maximum planned dose of 15 mg/kg of bevacizumab, 46 additional patients were treated at that dose level. Forty-eight percent of the patients had received prior chemotherapy for recurrent/metastatic disease. The combination was well tolerated. Three patients (5%) experienced serious bleeding events. The observed response rate was 15% with 4 complete responses allowing rejection of the null hypothesis that the response rate is ≤5% and the percentage of patients not progressing within two months is ≤30%. The median OS and PFS were 7.1 and 4.1 months, respectively. Higher ratios of phosphorylated over total VEGF receptor-2 and EGFR in pretreatment biopsies were associated with complete response (*P* = 0.036 and *P* = 0.036, resp.) and tumor shrinkage (*P* = 0.007 and *P* = 0.008, resp.) in a subset of 11 subjects with available tissue [102].

The feasibility and efficacy of adding bevacizumab and erlotinib to concurrent CRT in patients with LA SCCHN was evaluated in a phase II trial conducted by the Sarah Cannon oncology research consortium including 60 previously untreated patients [103]. The treatment consisted of induction chemotherapy with 6 weeks of paclitaxel, carboplatin, infusional 5-fluorouracil, and bevacizumab, which was followed by radiation therapy, weekly paclitaxel, bevacizumab, and erlotinib. After a median followup of 32 months, the estimated 3-year PFS and OS rates are 71% and 82%, respectively. Grade 3/4 mucosal toxicity occurred frequently (88%) during combined modality. No unexpected toxicity resulted from the addition of bevacizumab and erlotinib.

Integrins promote and regulate endothelial cell proliferation, migration, invasion, and survival in tumors, securing vascularisation and vascular remodeling in tumors. Cilengitide is a selective inhibitor of the *α*v*β*3/5 integrins. ADVANTAGE [104] is a phase I/II trial evaluating cilengitide in combination with cetuximab, cisplatin and 5-fluorouracil in patients with R/M SCCHN. No DLTs were observed in the phase I part of the study which tested cilengitide (500, 1000, and 2000 mg) twice weekly with standard doses of cetuximab, cisplatin and 5-fluorouracil. Cilengitide 2000 mg was considered safe and was selected for the subsequent randomised phase II part assessing PFS [104].

## 6. Conclusion

The road from preclinical evidence to clinical use is long and bumpy, and a large number of targeted agents are still at the start of the race. Some others reached the last stretch but stumbled on one of the last hurdles in phase II, or even phase III trials. Thus far, only the EGFR-directed monoclonal antibody cetuximab has made it to the finish and is currently approved for the treatment of locoregionally advanced and recurrent/metastatic SCCHN, by the regulatory agencies of the United States and Europe.

## Figures and Tables

**Table 1:** Planned or ongoing trials with cetuximab in SCCHN.

Trial phase	Disease setting	Associated treatment	Comparator
Phase III	LA resectable	TP before surgery	Surgery + RT
	LA	RT (after TPF ICT)	RT + P
	LA	RT after TPF	RT + P or CRT
	LA HPV + OPH	RT	P
	LA resected	RT	none

Randomized phase II	LA	RT	P
	LA	TPF or paclitaxel + carbo	
	LA	Adjuvant after CRT + cetuximab	none
	R/M	Temsirolimus	
	LA ICT	TPF or paclitaxel/P	

Single arm phase II	LA	RT + (P or carbo) + NABP	
	LA	(Accelerated) RT	
	LA ICT	TPF	
	LA ICT	TP	
	LA ICT	Paclitaxel/P → RT	
	LA after TPF ICT	RT	
	LA resected	CRT	
	LA after carbo/paclitaxel ICT	RT + P	
	Adjuvant after CRT	None	
	Local recurrence	BNCT	
	Local recurrence	RT	
	Local recurrence	RT + P	
	Local recurrence	Radiosurgery	
	Reirradiation	RT	
	R/M	carbo/P + pemetrexed	
	R/M	TP	
	R/M 2nd	carbo + vinorelbine	

LA: locoregionally advanced; R/M: recurrent/metastatic; RT: radiotherapy; P: cisplatin.

T: docetaxel; F: 5-fluorouracil; carbo: carboplatin; ICT: induction chemotherapy.

2nd: second line; NABP: nanoparticle albumin-bound paclitaxel.

BNCT: boron neutron capture therapy; CRT: concurrent chemoradiation; OPC: oropharyngeal cancer.

**Table 2:** Targeted agents under investigation in combination with cetuximab.

Trial phase	Associated Compound	Target/mechanism of action	Disease setting	Administration	Associated treatment
Randomized phase III	With or without 0S1-906	IGF-1R/IR	R/M platinum refractory	po	
	With or without E7050	VEGFR-2; c-MET	R/M platinum refractory	po	
	With or without bevacizumab	VEGF	LA	iv	Pemetrexed + RT
	With or without everolimus	mTOR	LA	po	Paclitaxel + P IC

Single arm phase II	Sorafenib	CRAF; BRAF; c-KIT; FLT-3; VEGFR-2/3; PDGFR-*β*	R/M	po	
	Temsirolimus	mTOR	R/M	iv	P
	PX-866	Phosphoinositide-3-kinase	R/M	po	
	Everolimus	mTOR	R/M	po	Carbo
	Bevacizumab	VEGF	R/M	iv	
	Bevacizumab	VEGF	LA	iv	(TP) → RT + P
	EMD1201081	Toll-like receptor 9 agonist	R/M 2nd	sc	
	Lenalidomide	Immunomodulating agent	R/M; solid tumors	po	
	Dasatinib	BCR-ABL; Src; c-KIT; EPH; PDGFR*β*	LA	po	RT (±P)
	BMS-754807	IGF-1R/IR	solid tumors	po	
	Erlotinib	EGFR	R/M	po	carbo + paclitaxel
	EGFR Antisense DNA	EGFR		Intratumoral	RT

Phase l	Everolimus	mTOR	LA	po	
	Lenalidomide	Immunomodulating agent	R/M	po	RT + P
	IPI-926	Smoothened, hedgehog pathway	R/M	po	
	VTX-2337	Toll-like receptor 8 agonist	R/M	sc	
	MM-121	ErbB3	Advanced tumors	iv	Irinotecan
	Lapatinib	EGFR/HER2	Solid tumors	po	
	RO5479599*	HER-3	HER-3 +tumors	iv	
	Sunitinib	PDGFR*α*/*β*; VEGFRl-3; KIT; FLT3; CSF-1R; RET	Local recurrence	po	RT
	Vorinostat	HDACi	LA	po	RT + P

mTOR: mammalian target of rapamycin; LA: locoregionally advanced; po: per os; iv: intravenously; RT: radiotherapy; EGFR: epidermal growth factor receptor;

P: cisplatin; carbo: carboplatin; T: docetaxel; R/M: recurrent/metastatic; OPH: oropharyngeal cancer; c-MET: hepatocyte growth factor receptor;

HDACi: histone deacetylase inhibitor; IGF-1R/IR: insulin-like growth factor-l/insulin receptor.

PDGFR*β*: platelet-derived growth factor recpetor *β*; BRAF: serine/threonine-protein kinase B-Raf; c-KIT: mast/stem cell growth factor receptor.

*: RO5479599 a lone or with erlotinib or with cetuximab.

**Table 3:** Targeted agents under investigation in SCCHN.

Trial phase	Compound	Target/mechanism of action	Disease setting	Administration	Associated treatment
Randomized phase lll	INGN 201	p53 gene	R/M	iv	PF
	Bevacizumab	VEGF	R/M	iv	platin-based doublet
	Reovirus serotype 3 dearing	Virus	R/M platinum refractory	iv	Placebo

Phase ll	Temsirolimus	mTOR	R/M	iv	Carbo + paclitaxel
	PX-866	Phosphoinositide-3-kinase	Solid tumors	po	T
	Everolimus	mTOR	R/M	po	(T)
	Everolimus	mTOR	LA	po	Carbo + paclitaxel
	BBI608	Cancer stem cells	Advanced malignancies	po	Paclitaxel
	Sorafenib	CRAF; BRAF; c-KIT; FLT-3; VEGFR-2/3; PDGFR-*β*	R/M	po	
	Cediranib	VEGFR-2/3	R/M	po	
	Tadalafil	Phosphodiesterase-5 inhibitor	R/M	po	
	Fostamatinib	syk	Solid tumors	po	
	LY2523355	Mitotic kinesin Eg5 inhibitor	Solid tumors	iv	
	Vorinostat	HDACi	R/M	po	Capecitabine
	Gossypol	Bcl-2	R/M	po	T
	Pazopanib	VEGFRl-3; PDGFR*α*/*β*; FGFR1,3,4; KIT; RET	R/M	po	
	ACE-041	ALK1	R/M second line	sc	
	Axitinib	VEGFRl-3; c-KIT; PDGFR	R/M	po	
	Dacomitinib	Pan-ERBB inhibitor	R/M platinum refractory	po	

Phase l	MAGE-A3/HPV 16	Vaccine	R/M		
	MLN9708	Proteasome inhibitor	Solid tumors	po	
	4SC-205	Mitotic kinesin Eg5 inhibitor	Advanced malignancies	po	
	SAR566658	HuDS6 + DM4*	DS6-positive tumors	iv	
	MEHD7945A	EGFR/HER3	Epithelial tumors	iv	
	CUDC-101	HDACi; EGFR; HER2	LA	po	RT + P

RT: radiotherapy; T: docetaxel; P: cisplatin; HDACi: histone deacetylase inhibitor; LA: locoregionally advanced; R/M: recurrent/metastatic.

*Tumor-associated sialoglycotope CA6 (huDS6) conjugated to the cytotoxic maytansinoid DM4.

Syk: spleen tyrosine kinase; po: per os; iv: intravenously.

ALK1: activin receptor-like kinase 1 (ALK1).

**Table 4:** EGFR-directed targeted agents under investigation in SCCHN (other than cetuximab).

Trial phase	Compound	Disease setting	Associated treatment	Comparator
Phase III	Monoclonal Antibodies			
	Nimotuzumab	LA resected	RT + P	Placebo
		LA	RT	None
	Zalutumumab	nonmetastatic	RT (±P)	None
	Tyrosine kinase inhibitors			
	Lapatinib*	resected high risk		Placebo
	Afatinib*	LA and NED after RT + P (+S)		Placebo
		LA after resection and RT + P		Placebo
		R/M platinum-refractory		Methotrexate

Randomized phase II	Tyrosine kinase inhibitors			
	Erlotinib	LA	RT + P	None
	Erlotinib	R/M	TP	Placebo

Phase II	Monoclonal antibodies			
	Panitumumab	R/M	Paclitaxel	
	Nimotuzumab	LA	RT+ P	
		LA	(T) PF ICT	
	SYM-004***	R/M platinum-refractory		
	Tyrosine kinase inhibitors			
	Lapatinib*	LA	RT	
	Lapatinib*	R/M	Capecitabine	
	Erlotinib	LA	RT (+T)	
		LA resected	RT+ P	
		Local recurrence/2nd primary	RT + pemetrexed	
		Local recurrence		

Phase I	Monoclonal antibodies			
	ABT-806**	Solid tumors		
	R05083945 or cetuximab	Operable		

NED: no evidence of disease; S: lymph node resection.

CRT: concurrent chemoradiation; ICT: induction chemotherapy.

T: docetaxel; P: cisplatin.

*Dual EGFR/HER2 inhibitor. **Targets also EGFRvIII. ***Recombinant IgG1 antibody product consisting of two antibodies against EGFR.
